# PCIF1 Attenuates Type I Interferon Induction by Inhibiting IRF3 Activation in a Methyltransferase-Independent Manner

**DOI:** 10.3390/cells15030303

**Published:** 2026-02-05

**Authors:** Ryoya Kano, Chihiro Oyama, Chihiro Ikeda, Akiko Inujima, Keiichi Koizumi, Shinichiro Akichika, Tsutomu Suzuki, Aki Tanaka, Yoshiaki Ohkuma, Yutaka Hirose

**Affiliations:** 1 Department of Gene Regulation, Faculty of Pharmaceutical Sciences, University of Toyama, 2630 Sugitani, Toyama 930-0194, Japan; d2362303@ems.u-toyama.ac.jp (R.K.); m24b1312@ems.u-toyama.ac.jp (C.O.); m25b1302@ems.u-toyama.ac.jp (C.I.); atanaka@pha.u-toyama.ac.jp (A.T.); ohkumay@mocha.ocn.ne.jp (Y.O.); 2 Department of Medical Oncology, Kanazawa Medical University, 1-1 Daigaku, Uchinada-Machi, Kahoku 920-0293, Japan; inujima@kanazawa-med.ac.jp; 3 Division of Presymptomatic Disease, Institute of Natural Medicine, University of Toyama, Toyama 930-0194, Japan; kkoizumi@inm.u-toyama.ac.jp; 4 Department of Chemistry and Biotechnology, Graduate School of Engineering, The University of Tokyo, Tokyo 113-8656, Japan; sakichika@chembio.t.u-tokyo.ac.jp (S.A.); tomsuzuki@g.ecc.u-tokyo.ac.jp (T.S.); 5 Department of Biochemistry, Graduate School of Biomedical Sciences, Nagasaki University, 1-12-4 Sakamoto, Nagasaki 852-8523, Japan

**Keywords:** innate immunity, antiviral response, type I interferon, transcriptional regulation, mRNA modifications, m^6^Am, PCIF1, IRF3

## Abstract

PCIF1 is primarily recognized as an RNA methyltransferase that mediates *N*^6^-methylation of cap-proximal adenosine (m^6^Am) and plays diverse roles in gene expression. In this study, we uncover a novel role for PCIF1 as a crucial negative regulator of type I interferon (IFN) induction, a pathway critical for antiviral immunity whose dysregulation leads to inflammatory and autoimmune diseases. We demonstrate that PCIF1 deficiency robustly enhances the poly(I:C)-induced type I IFN response, accompanied by augmented STAT1 activation and interferon-stimulated gene (ISG) expression. Mechanistically, PCIF1 suppresses *IFNB1* transcription by attenuating IRF3 phosphorylation and nuclear translocation, as shown by increased nascent *IFNB1* mRNA synthesis and promoter activity in PCIF1-deficient cells, without affecting the mRNA stability. Crucially, this suppressive function was independent of PCIF1’s canonical RNA methyltransferase activity, as both wild-type PCIF1 and a methyltransferase-inactive mutant effectively attenuated type I IFN induction. Collectively, our findings establish PCIF1 as a novel methyltransferase-independent suppressor of type I IFN responses, revealing its previously unrecognized non-catalytic function. This discovery offers critical insights into the multifaceted regulation of innate immunity and highlights PCIF1’s non-catalytic function as a promising therapeutic target for modulating antiviral responses and inflammatory diseases.

## 1. Introduction

The innate immune system is the first line of defense against pathogen invasion [1]. Recognition of invading pathogens by host pattern recognition receptors (PRRs) that detect pathogen-associated molecular patterns (PAMPs) triggers intracellular signaling cascades that activate gene expression programs involved in innate immune responses [1,2]. In particular, during RNA virus infection, host sensors such as Toll-like receptor 3 (TLR3) and retinoic acid-inducible gene I-like receptors (RLRs) detect viral RNAs and activate downstream signaling pathways [2,3,4]. This signaling induces the production of inflammatory cytokines and type I interferons (IFNs) through the activation of key transcription factors such as NF-κB and IRF3 [5,6]. Secreted type I IFNs bind to cognate receptors and activate the JAK–STAT pathway, leading to the widespread expression of interferon-stimulated genes (ISGs), which function as critical antiviral effectors [7,8]. However, excessive activation or loss of homeostasis in the type I IFN response pathway can lead to inflammatory and autoimmune diseases [9,10]. Therefore, proper regulation of the innate immune response and maintenance of homeostasis are crucial at the host defense–disease interface.

Recently, epitranscriptomes have emerged as a critical layer influencing antiviral immune responses, including the type I IFN pathway [11]. Among these, *N*^6^-methyladenosine (m^6^A), the most abundant internal modification in eukaryotic mRNA, plays multifaceted roles in host–virus interactions [12,13,14]. For instance, the presence of m^6^A on interferon-β (IFN-β) mRNA has been reported to suppress the antiviral response upon viral infection [15,16], and m^6^A introduced into viral RNA by host methyltransferases METTL3 and METTL14 may contribute to the evasion of viral nucleic acid recognition by RIG-I [17,18].

In addition to internal m^6^A, *N*^6^,2′-*O*-dimethyladenosine (m^6^Am), a specific modification at the *N*^6^ position of the cap-proximal adenosine, has emerged as another prevalent epitranscriptomic mark in vertebrates [19]. m^6^Am is thought to be functionally different from internal m^6^A, and its *N*^6^ methylation is catalyzed by the novel RNA methyltransferase PCIF1(CAPAM) [20,21,22,23]. Although the precise role of m^6^Am in gene expression regulation remains debated, PCIF1-knockout studies suggest its involvement in regulating mRNA transcription, stability, and translation, thereby influencing various biological processes such as stress response [20,24], metabolism [25,26,27], cancer [28,29,30,31,32,33], and immune processes [34,35,36,37].

PCIF1 is primarily recognized for its catalytic function; however, recent investigations suggest that PCIF1 may exhibit functions independent of its methyltransferase activity [28,38,39]. We initially identified PCIF1 as a protein that interacts with the phosphorylated C-terminal domain (CTD) of RNA polymerase II (Pol II) through its WW domain [40]. Furthermore, we previously demonstrated that PCIF1 significantly inhibits the activation of reporter gene expression driven by the activation domains of various transcription factors, with this reduction largely dependent on the CTD-binding capacity of the WW domain [41]. Therefore, PCIF1 may possess functions, particularly through its WW domain, that are independent of its catalytic activity, although these functions remain largely unexplored.

Intriguingly, recent studies have reported that PCIF1-mediated m^6^Am formation is involved in controlling viral infections, exhibiting both proviral and antiviral effects linked to its m^6^Am-depositing activity [34,35,36]. For instance, PCIF1 has been implicated in suppressing HIV transcription [35] and promoting coronavirus infections by stabilizing host receptors [36]. Notably, Tartell et al. demonstrated that PCIF1 modifies several RNA viruses with m^6^Am, although these modifications have no effect on viral replication and infectivity. However, PCIF1 could attenuate IFN-β-mediated antiviral effects through its methyltransferase activity [34]. While these studies highlight PCIF1’s role in host–virus interactions, they predominantly focus on m^6^Am-dependent mechanisms and offer limited insights into PCIF1’s direct influence on the initial induction of type I IFN responses. Furthermore, the critical question of whether PCIF1’s canonical methyltransferase activity is exclusively responsible for its role in IFN induction remains unanswered.

In this study, we initially reexamined our previous data by genome-wide transcriptional analysis in PCIF1-knockdown cells [42] and found a significant enrichment of type I IFN-related genes among the upregulated transcripts. This observation suggests an uncharacterized negative regulatory role for PCIF1 in the type I IFN response. While a previous study hinted at a suppressive role of PCIF1 in IFN-β activity during specific viral infections [34], the molecular mechanisms underlying its suppressive function, including the specific genes and signaling processes that PCIF1 regulates, remain unexplored. Therefore, we aimed to elucidate the precise molecular mechanisms by which PCIF1 regulates the induction of type I IFN responses. Our findings reveal that PCIF1 acts as a novel suppressor of type I IFN induction through a mechanism independent of its canonical methyltransferase activity. Thus, this study provides critical insights into the multifaceted regulation of innate immunity and identifies PCIF1 as a potential therapeutic target for modulating antiviral responses.

## 2. Materials and Methods

### 2.1. Cell Culture

All cells were maintained at 37 °C in a humidified atmosphere containing 5% CO_2_. HeLa S3 cells were cultured in Dulbecco’s Modified Eagle’s Medium (DMEM) (Shimazu Diagnostics Corporation, Tokyo, Japan) supplemented with 5% calf serum (Cytiva, Tokyo, Japan), 1% penicillin/streptomycin, and 2 mM of L-glutamine. 293T and A549 cells were cultured in DMEM supplemented with 10% fetal bovine serum (Nichirei Biosciences Inc., Tokyo, Japan), 1% penicillin/streptomycin, and 2 mM of L-glutamine.

### 2.2. Generation of PCIF1-Knockout (KO) 293T and A549 Cells

PCIF1-knockout (KO) 293T cells were established using the CRISPR-Cas9 system as previously described [20]. Similarly, PCIF1 KO A549 cells were established following the same protocol applied to the PCIF1 KO 293T cells, employing the identical sgRNA sequence that targets exon 4 of PCIF1. The sgRNA sequence is specified as follows: 5′-CGA TTC ACC AAC CAG TCC CTG-3′. The generation of PCIF1 KO clones was confirmed through immunoblotting.

### 2.3. Cell Proliferation Assay

WT and PCIF1 KO A549 cells were seeded in 96-well plates at a density of 2.0 × 10^3^ cells per well. Each day, 1/10 volume of 1 mM resazurin solution (Tokyo Chemical Industry Co., Ltd., Tokyo, Japan) in phosphate-buffered saline (PBS) was added to each well, and the cells were incubated for 1 h. The absorbance of reduced resazurin was measured using the iMark™ Microplate Absorbance Reader (Bio-Rad Laboratories, Inc., Hercules, CA, USA) at 570 nm.

### 2.4. Small Interfering RNA (siRNA) Transfection

For siRNA-mediated PCIF1 knockdown, cells were transfected with siRNA (20 nM) using Lipofectamine™ RNAiMAX Transfection Reagent (Thermo Fisher Scientific, Waltham, MA, USA) for 48 h. The siRNA sequences are listed in Supplementary Document S1.

### 2.5. Analyses of DNA Microarray Data

DNA microarray analyses of PCIF1-knockdown HeLa cells treated with two different siRNAs were performed as previously described (accession number GSE156768) [42]. The differentially expressed genes (DEGs) that showed a two-fold or greater change in expression upon PCIF1 knockdown were identified using the GENESPRING software version 11.0 (Agilent Technologies, Inc., Santa Clara, CA, USA). Among these, 78 downregulated and 112 upregulated genes were common to each siRNA-treated HeLa cell line. The identified DEGs were visualized in a volcano plot using R software version 4.5.1. The upregulated genes were subjected to Reactome, https://reactome.org/ (accessed on 21 September 2021), version 77 to analyze significant pathways.

### 2.6. Cell Treatment

Cells were seeded in 12-well plates at a density of 1.5 × 10^5^ cells per well and typically cultured at 37 °C for 24 h before stimulation. For stimulation with poly(I:C) (Sigma-Aldrich Japan Inc., Tokyo, Japan), the cells were transfected with 0.1 μg/mL poly(I:C) using the Lipofectamine™ RNAiMAX Transfection Reagent. Lipopolysaccharide (LPS) and R848 were kindly provided by Dr. O. Takeuchi (Department of Medical Chemistry, Graduate School of Medicine, Kyoto University, Kyoto, Japan). For stimulation with LPS or R848, the cells were treated with 50 μg/mL of LPS or 20 μM of R848. To induce a type I interferon response, the cells were treated with 20 ng/mL of IFN-β (Proteintech Group, Inc., Rosemont, IL, USA).

### 2.7. Plasmid Construction and Transfection

To generate a Flag-tagged PCIF1 expression vector, a complementary DNA (cDNA) fragment encoding full-length PCIF1 (NCBI accession no. NM_022104) was amplified by PCR from a cDNA pool synthesized from HeLa cell total RNA using the following primer set: 5′-TTCGAATTCGATGGCCAATGAGAATCACGGCAG-3′ and 5′-CGACTCGAGTTAAGTGGGGTGAGGCTCGCGGCT-3′. In addition, the fragment was cloned into a pcDNA3-FLAG vector (Thermo Fisher Scientific). The catalytically inactive PCIF1 mutant (PCIF1_NAAF_) vector was generated using the KOD-Plus Mutagenesis Kit (TOYOBO Co., Osaka, Japan) by inverse PCR using the pcDNA3-FLAG-PCIF1 vector as a template with the following mutagenic primers: 5′-GCCGCCTGCGAGGAGCTCATGGATGCCA-3′ and 5′-AGGGTTGGCCTCAAATGAACCACTC-3′. All PCR reactions were performed using KOD-Plus DNA polymerase (TOYOBO) according to the manufacturer’s instructions. All sequences derived from PCR amplification were verified by DNA sequencing. HEK293T cells were transfected with DNA using a polyethyleneimine (PEI) reagent.

### 2.8. Immunoblotting

The cells were washed with ice-cold PBS and then lysed in 100 μL of radioimmunoprecipitation assay (RIPA) buffer [50 mM of Tris-HCl (pH 7.9), 150 mM of NaCl, 1% NP-40, 0.1% SDS, 0.5% sodium deoxycholate, and 1 mM of EDTA with the protease inhibitor cocktail]. The cell lysates were subjected to SDS-PAGE, followed by electroblotting onto PVDF membranes (FUJIFILM Wako Pure Chemical Corporation, Osaka, Japan). Membranes were blocked with 2% bovine serum albumin (BSA) and then incubated with the indicated primary and HRP-conjugated secondary antibodies. The proteins were detected using the Chemi-Lumi One L (NACALAI TESQUE, Inc., Kyoto, Japan) on ImageQuant^TM^ LAS 4000 mini (Cytiva).

### 2.9. Immunofluorescence

A549 cells cultured on coverslips were fixed with 4% formaldehyde (Thermo Fisher Scientific) in PBS for 20 min and permeabilized with 0.5% Triton X-100 in PBS for 10 min. After washing with PBS, the cells were blocked with 1% bovine serum albumin (BSA) for 30 min. The cells were incubated with the primary antibody in PBS containing 1% BSA at 4 °C overnight, followed by incubation with the appropriate secondary antibody in PBS containing 1% BSA at room temperature for 45 min. Coverslips were mounted on glass slides with the VECTASHIELD Mounting Medium with DAPI (Vector Laboratories, Inc., Newark, CA, USA), and images were captured using the Zeiss LSM 700 confocal laser scanning microscope (Zeiss, Jena, Germany). The images were processed and analyzed using the Fiji software version 1.54p.

### 2.10. Reverse Transcription Quantitative PCR (RT-qPCR)

Total RNA was isolated from the cells using Sepasol™-RNA I Super G (NACALAI TESQUE, Tokyo, Japan) according to the manufacturer’s instructions. First-strand cDNA was synthesized from total RNA using the ReverTra Ace^TM^ qPCR RT Master Mix with gDNA Remover (TOYOBO, Osaka, Japan) with random hexamer primers, according to the manufacturer’s instructions. cDNA was quantified using the GeneAce SYBR^TM^ qPCR Mix II (NIPPON GENE Co., Tokyo, Japan) and the Mx3000P real-time PCR system (Agilent Technologies). Relative RNA expression levels were calculated using the 2^−ΔΔCt^ method and normalized to 18S rRNA.

### 2.11. Nascent RNA Analysis

Nascent RNAs were captured using the Click-iT Nascent RNA Capture Kit (Thermo Fisher Scientific). A549 cells were stimulated with poly(I:C) for 6 h and treated with 5-ethynyl uridine (5-EU) (200 μM) to label nascent RNAs. Total RNA was isolated from cells using Sepasol™-RNA I Super G (NACALAI TESQUE) according to the manufacturer’s instructions. EU-labeled RNA was biotinylated with 0.5 mM of biotin azide by the click reaction, and the biotinylated RNA was purified using streptavidin beads. For cDNA synthesis, the ReverTra Ace^TM^ qPCR RT Master Mix with gDNA Remover (TOYOBO) was used. cDNA was quantified using the GeneAce SYBR^TM^ qPCR Mix II (NIPPON GENE) and the Mx3000P real-time PCR system. Relative RNA expression levels were calculated using the 2^−ΔΔCt^ method and normalized to 18S rRNA.

### 2.12. Dual-Luciferase Assay

To determine the effect of PCIF1 depletion on the promoter activity of IFN-β induced by poly(I:C), WT and PCIF1 KO 293T cells were seeded on 24-well plates at a density of 5 × 10^4^ cells per well and typically cultured at 37 °C for 24 h before transfection. The cells were co-transfected with 100 ng of *Firefly* luciferase reporter plasmid IFN-β-Luc [kindly provided by Dr. O. Takeuchi (Department of Medical Chemistry, Graduate School of Medicine, Kyoto University, Kyoto, Japan)] and 5 ng of internal control *Renilla* luciferase vector phRG-B (Promega Corporation, Madison, WI, USA) for 24 h. After transfection, the cells were stimulated with poly(I:C) for 6 h, and luciferase assays were performed using the Dual-Luciferase Assay System (Promega) according to the manufacturer’s instructions.

### 2.13. mRNA Stability Assay

To assess mRNA stability, 5 µg/mL actinomycin D (Sigma-Aldrich) was added to A549 cells after 6 h of poly(I:C) transfection to inhibit transcription. Cells were harvested at 0, 2, 4, 6, and 8 h after actinomycin D treatment, and total RNA was isolated from each time point. The amount of remaining *IFNB1* mRNA was measured using RT-qPCR. Relative values were calculated using the expression level of β-actin mRNA (*ACTB*) as a normalizer.

### 2.14. Statistical Analysis

All data are presented as means ± standard deviation (SD) from at least three independent experiments. A two-tailed *t*-test and two-way ANOVA with Tukey’s test were performed to determine statistical significance. Statistical significance is indicated by asterisks in the figures [not significant (n.s.), *p* > 0.05; * *p* < 0.05; ** *p* < 0.01; *** *p* < 0.001].

## 3. Results

### 3.1. PCIF1 Negatively Regulates the Expression of a Subset of Interferon-Stimulated Genes

**Figure 1:**
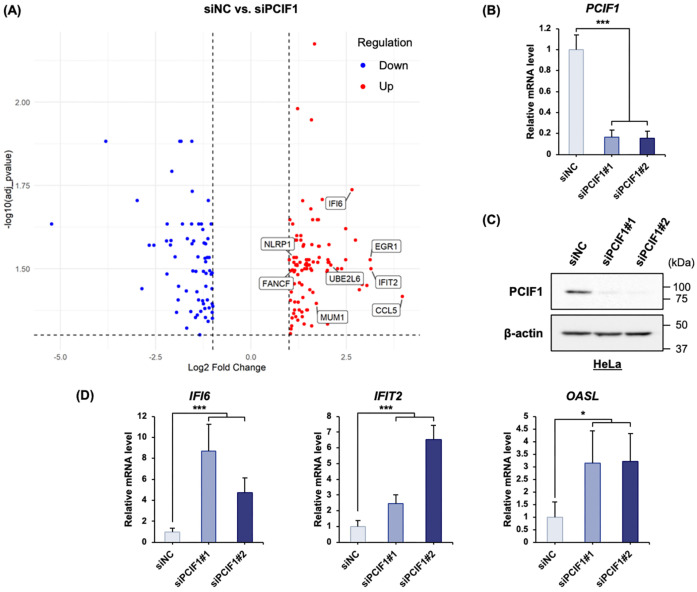
PCIF1 negatively regulates the expression of a subset of interferon-stimulated genes. (**A**) A volcano plot is employed to illustrate the 190 DEGs identified through DNA microarray analysis of HeLa cells treated with siRNA, using thresholds of log2 fold change > 0.5 and an adjusted *p*-value < 0.05. Genes with increased expression are depicted in red, and those with decreased expression are shown in blue. Genes associated with the type I IFN pathway are indicated by a square box. (**B**–**D**) HeLa cells were treated with negative control siRNA (siNC) or two distinct siRNAs targeting PCIF1 (siPCIF1#1 and #2) for 72 h. (**B**) RT-qPCR analysis of total RNA isolated from siRNA-treated HeLa cells using a specific primer set for *PCIF1* mRNA. (**C**) Immunoblot analysis of total protein extracts from siRNA-treated HeLa cells using anti-PCIF1 and anti-β-actin antibodies. (**D**) RT-qPCR analysis of total RNA isolated from siRNA-treated HeLa cells using specific primer sets for *IFI6*, *IFIT2*, and *OASL* genes. Data are presented as mean ± SD from three independent experiments. Statistical significance was determined using a two-tailed *t*-test (* *p* < 0.05; *** *p* < 0.001).

We previously performed DNA microarray analyses in HeLa cells transfected with either control or two different PCIF1-targeting siRNAs and identified 190 differentially expressed genes (DEGs) that showed a two-fold or greater change in expression upon PCIF1 knockdown (Supplemental Table S1) [42]. These DEGs were visualized in a volcano plot using the thresholds of log2 fold change > 0.5 and adjusted *p*-value < 0.05 (Figure 1A). The upregulated 112 DEGs were subjected to pathway analysis using the Reactome database [43], which revealed a significant enrichment of genes involved in “interferon alpha/beta signaling” and “interferon signaling” pathways (Table 1, Figure 1A). To validate these findings, we focused on three representative ISGs (*IFI6*, *IFIT2*, and *OASL*) that showed robust upregulation in the microarray data. Transfection of HeLa cells with two independent siRNAs targeting PCIF1 (siPCIF1#1 and siPCIF1#2) effectively suppressed *PCIF1* mRNA and protein levels (Figure 1B,C). Consistent with the DNA microarray results, the mRNA expression of *IFI6*, *IFIT2*, and *OASL* was markedly increased in PCIF1-knockdown cells compared to that in cells treated with control siRNA (Figure 1D), indicating that PCIF1 suppresses the mRNA expression of a subset of ISG. Based on these results, we hypothesized that PCIF1 is involved in the type I IFN pathway by negatively regulating the expression of a subset of interferon regulatory genes.

The upregulated 112 DEGs were subjected to Reactome version 77 to analyze significant pathways. The table shows the ten most relevant pathways sorted by *p*-value.

### 3.2. PCIF1 Deficiency Enhances the dsRNA-Induced STAT1 Activation and ISG Expression in 293T Cells

**Figure 2:**
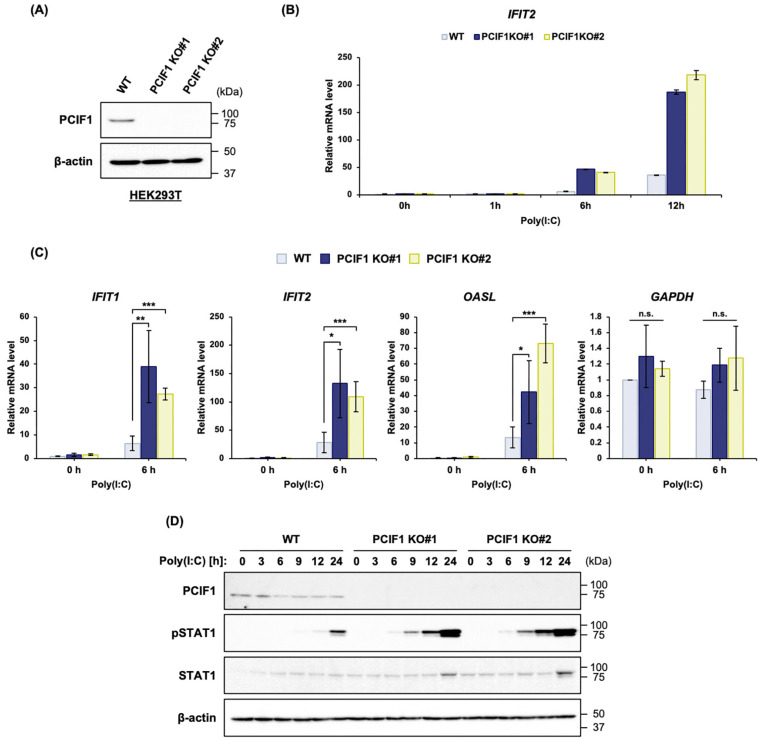
PCIF1 deficiency promoted dsRNA-induced STAT1 activation and ISG expression in 293T cells. (**A**) Immunoblot analysis of total protein extracts from WT and two different PCIF1 KO 293T cells using anti-PCIF1 and anti-β-actin antibodies. (**B**–**D**) WT and PCIF1 KO (#1 and #2) 293T cells were stimulated with poly(I:C). The cells were harvested at each time point after transfection with poly(I:C). (**B**) RT-qPCR analysis of total RNA isolated from WT and PCIF1 KO (#1 and #2) HEK293T cells using a specific primer set for *IFIT2* mRNA. (**C**) RT-qPCR analysis of total RNA isolated from WT and PCIF1 KO (#1 and #2) 293T cells using a specific primer set for *IFIT1*, *IFIT2*, *OASL*, and *GAPDH* mRNAs. (**D**) Immunoblot analysis of total protein extracts from poly(I:C)-stimulated 293T cells using the indicated antibodies. Data are presented as mean ± SD from three independent experiments. Statistical significance was determined using a two-tailed *t*-test (n.s., *p* > 0.05; * *p* < 0.05; ** *p* < 0.01; *** *p* < 0.001).

To further investigate the potential involvement of PCIF1 in type I IFN responses, we used a system in which type I IFN responses were induced by transfecting cells with polyinosine polycytidylic acid [poly(I:C)], a synthetic double-stranded RNA (dsRNA) analog of viral RNA, and examined the effect of PCIF1 deficiency on type I IFN responses. First, we examined whether poly(I:C) stimulation affects PCIF1 protein expression and found that poly(I:C) stimulation did not significantly affect PCIF1 expression (Figure S1A,B). Next, using two independently generated PCIF1-knockout (KO) human 293T cell lines (KO #1 and KO #2) [20] and their parental wild-type (WT) counterpart (Figure 2A), we investigated the impact of PCIF1 deficiency on type I IFN responses. Both PCIF1 KO lines consistently showed markedly enhanced *IFIT2* expression following poly(I:C) stimulation compared to WT cells (Figure 2B). Similarly, RT-qPCR analysis revealed a marked increase in the expression of other ISGs (*IFIT1* and *OASL*) in both PCIF1KO cell lines, whereas *GAPDH* expression remained unaffected (Figure 2C). Since the induction of ISGs requires the activation of the transcription factor STAT1 [7,44], we next assessed the effect of PCIF1 deficiency on STAT1 activation by evaluating phosphorylation at Tyr701, a marker of activation [44]. Immunoblotting analysis using a phospho-specific antibody showed that poly(I:C)-induced STAT1 Tyr701 phosphorylation in PCIF1 KO cells was detectable much earlier than in WT cells (see 6 h) and reached significantly higher levels at 24 h than in WT cells (Figure 2D). These results suggest that PCIF1 suppresses dsRNA-induced ISG expression by inhibiting STAT1 activation.

### 3.3. PCIF1 Deficiency Promoted the dsRNA-Induced STAT1 Activation and ISG Expression in A549 Cells

**Figure 3:**
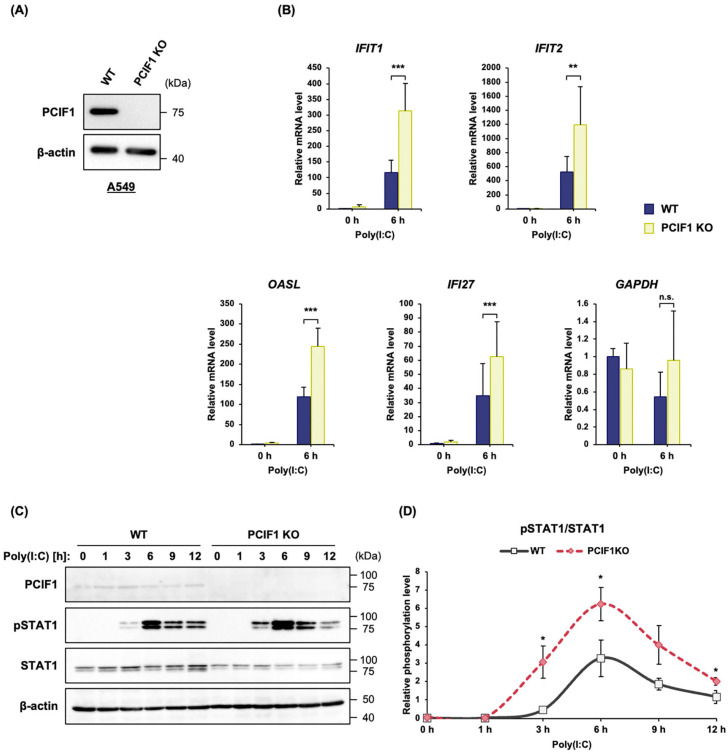
PCIF1 deficiency promoted dsRNA-induced STAT1 activation and ISG expression in A549 cells. (**A**) Immunoblot analysis of total protein extracts from WT and PCIF1 KO A549 cells using anti-PCIF1 and anti-β-actin antibodies. (**B**) WT and PCIF1 KO A549 cells were stimulated with poly(I:C) for 6 h. RT-qPCR analysis of total RNA isolated from WT and PCIF1 KO A549 cells using a specific primer set for *IFIT1*, *IFIT2*, *OASL*, *IFI27*, and *GAPDH* mRNAs. (**C**) A549 cells were stimulated with poly(I:C), and the cells were harvested at different time points after stimulation. Immunoblot analysis of total protein extracts from WT and PCIF1 KO A549 cells using the indicated antibodies. (**D**) Signal intensities obtained from the immunoblots were quantified using the Fiji software version 1.54p. The intensities of pSTAT1 were normalized to that of the total STAT1. Data are presented as mean ± SD from three independent experiments. Statistical significance was determined using a two-tailed *t*-test (n.s., *p* > 0.05; * *p* < 0.05; ** *p* < 0.01; *** *p* < 0.001).

To ensure that the observed effects of PCIF1 deficiency were not cell type-specific and to broaden the physiological relevance of our findings, we extended our investigation to A549 cells, a human lung carcinoma cell line known for its strong type I IFN response and relevance to respiratory viral infections. We generated a PCIF1 KO A549 cell line using the CRISPR-Cas9 system. Immunoblotting analysis confirmed the absence of PCIF1 in this cell line (Figure 3A). As shown in Figure S2A, PCIF1 deletion in A549 cells did not alter the proliferation rate, similar to the observation in 293T cells [20]. The time course of STAT1 Tyr701 phosphorylation in A549 cells upon poly(I:C) stimulation showed that STAT1 phosphorylation was observed 3 h after poly(I:C) transfection, reaching its peak at 6 h (Figure S2B). In addition, poly(I:C) stimulation did not significantly affect PCIF1 protein levels (Figure S2B,C), similar to the observations in 293T cells. Next, we examined whether the ISG expression induced by poly(I:C) stimulation was affected in PCIF1 KO A549 cells using RT-qPCR. The results showed that the induction of ISG expression, including *IFIT1*, *IFIT2*, *OASL*, and *IFI27*, was markedly enhanced in PCIF1 KO A549 cells compared to that in WT cells (Figure 3B). In contrast, the expression of the constitutively expressed *GAPDH* was not affected by either poly(I:C) transfection or PCIF1 KO (Figure 3B). We also assessed the effect of PCIF1 deficiency on STAT1 activation in A549 cells using a phospho-specific antibody for immunoblotting. Similar to the results observed in 293T cells, poly(I:C)-induced phosphorylation of STAT1 Tyr701 in PCIF1 KO A549 cells occurred earlier and more robustly than in WT cells (Figure 3C,D).

**Figure 4:**
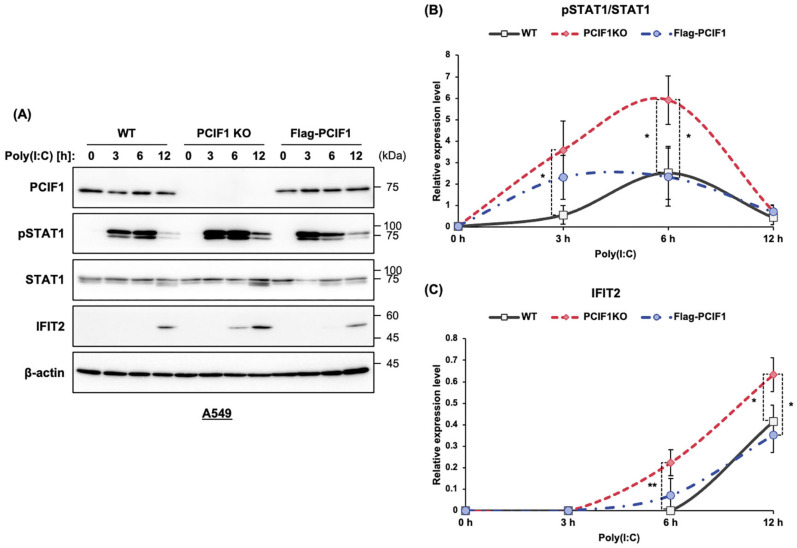
Ectopic expression of PCIF1 restored normal phosphorylation levels of STAT1 at Tyr701 upon poly(I:C) stimulation. (**A**) A549 cells were stimulated with poly(I:C), and the cells were harvested at different time points after stimulation. Immunoblot analysis of total protein extracts from WT, PCIF1 KO, and Flag-PCIF1 expressed in A549 cells using the indicated antibodies. (**B**,**C**) Signal intensities obtained from the immunoblots were quantified using the Fiji software version 1.54p. The intensities of pSTAT1 (**B**) and IFIT2 (**C**) were normalized to those of total STAT1 and β-actin, respectively. Data are presented as mean ± SD from three independent experiments. Statistical significance was determined using a two-tailed *t*-test (* *p* < 0.05; ** *p* < 0.01).

Next, to confirm that the enhanced type I IFN response observed in PCIF1-deficient cells was indeed due to the loss of PCIF1, we conducted rescue experiments by expressing wild-type PCIF1 in PCIF1 KO A549 cells. The cells were transfected with a construct expressing Flag-PCIF1 to restore PCIF1 expression to levels similar to those of the endogenous PCIF1. We analyzed STAT1 phosphorylation levels in response to poly(I:C) stimulation using immunoblotting. Expression of Flag-PCIF1 partially restored STAT1 phosphorylation 3 h after poly(I:C) transfection, and by 6 h, STAT1 phosphorylation was fully restored to WT levels (Figure 4A,B). Furthermore, we analyzed whether the enhanced induction of *IFIT2* expression due to PCIF1 loss could be rescued by the exogenous PCIF1 expression. Expression of Flag-PCIF1 in PCIF1 KO A549 cells restored the IFIT2 induction pattern to that of WT cells (Figure 4A,C). Collectively, these findings suggest that PCIF1 may suppress ISG expression by inhibiting poly(I:C)-induced STAT1 activation.

### 3.4. PCIF1 Deficiency Promoted dsRNA-Induced Type I Interferon Production

**Figure 5:**
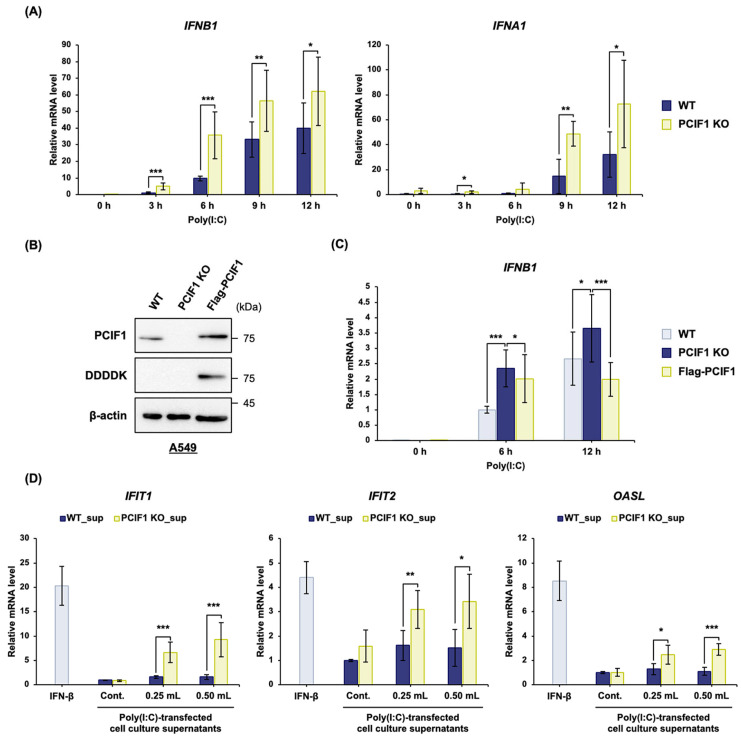
PCIF1 deficiency promoted dsRNA-induced type I interferon production. (**A**–**C**) A549 cells were stimulated with poly(I:C), and the cells were harvested at the indicated time points. (**A**) RT-qPCR analysis of total RNA isolated from WT and PCIF1 KO A549 cells using a specific primer set for *IFNB1* and *IFNA1* mRNAs. (**B**) Immunoblot analysis of total protein extracts from WT, PCIF1 KO, and Flag-PCIF1 stably expressing A549 cells using the indicated antibodies. (**C**) RT-qPCR analysis of total RNA isolated from WT, PCIF1 KO, and Flag-PCIF1 stably expressing A549 cells using a specific primer set for *IFNB1* mRNA. (**D**) WT and PCIF1 KO A549 cells were stimulated with poly(I:C), and the cell culture supernatants were harvested 6 h after stimulation. Supernatants or IFN-β (as a control) were added to WT A549 cells and incubated for 30 min, followed by RT-qPCR analysis of total RNA isolated from the cells using specific primer sets for *IFIT1*, *IFIT2*, and *OASL* mRNAs. Data are presented as mean ± SD from three independent experiments. Statistical significance was determined using a two-tailed *t*-test (* *p* < 0.05; ** *p* < 0.01; *** *p* < 0.001).

Given that PCIF1 deficiency robustly enhanced STAT1 activation and ISG expression in both 293T and A549 cells, we next sought to determine whether this effect stemmed from increased upstream type I IFN gene expression, a critical component that drives these downstream responses. We evaluated the mRNA expression levels of *IFNA1* and *IFNB1* in response to poly(I:C) stimulation in PCIF1 KO A549 cells using RT-qPCR. The induction of *IFNA1* and *IFNB1* expression following poly(I:C) stimulation was significantly enhanced in PCIF1 KO A549 cells compared to that in WT cells (Figure 5A). A marked enhancement in *IFNB1* mRNA induction was also observed in PCIF1 KO 293T cells (Figure S3), further supporting these findings. To further confirm that these response changes resulted from PCIF1 deficiency, we tested whether exogenous WT PCIF1 expression in PCIF1 KO A549 cells could restore *IFNB1* mRNA expression. First, we established a stable A549 cell line expressing Flag-PCIF1 at levels comparable to those of the endogenous PCIF1 (Figure 5B). We analyzed the induction of *IFNB1* mRNA expression following poly(I:C) stimulation using RT-qPCR. Expression of Flag-PCIF1 led to a partial recovery of *IFNB1* mRNA induction 6 h after poly(I:C) stimulation and a full recovery to WT levels after 12 h (Figure 5C). Because activation of the type I IFN signaling pathway is mediated by autocrine or paracrine processes involving secreted type I IFN [45], we examined whether the enhanced protein expression of type I IFN genes in PCIF1 KO cells led to enhanced induction of downstream ISG. In this study, we used the culture supernatants of WT and PCIF1 KO A549 cells after poly(I:C) stimulation to treat WT cells and investigated whether ISG induction occurred. The supernatant from PCIF1 KO A549 cells induced excessive dose-dependent expression of ISGs (*IFIT1*, *IFIT2*, and *OASL*) compared to that from WT cells (Figure 5D). In summary, induction of type I IFN gene expression by dsRNA stimulation is enhanced by PCIF1 deficiency, which in turn leads to increased downstream STAT1 phosphorylation and ISG expression levels.

### 3.5. PCIF1 Deficiency Promoted LPS or ssRNA-Induced Type I Interferon Production

Type I IFN responses are induced not only through dsRNA pathways involving TLR3 and RLRs (RIG-I and MDA5) but also through the recognition of other PAMPs [46]. TLR4 is activated by recognizing microbial components such as LPS, whereas TLR7/8 is activated by recognizing single-stranded RNA (ssRNA) molecules, triggering type I IFN and inflammatory responses [46]. Therefore, we investigated whether the enhancement in the type I IFN response via PCIF1 suppression could be observed in pathways other than the dsRNA pathway. As shown in Figure S4A, *IFNB1* mRNA expression was induced more rapidly and strongly in PCIF1 KO A549 cells than in WT cells following LPS stimulation. Next, when cells were stimulated with R848, an imidazoquinoline that specifically activates the TLR7/8 pathway, enhanced *IFNB1* mRNA expression was observed in PCIF1 KO A549 cells compared to that in WT cells (Figure S4B). These results suggest that PCIF1 negatively regulates the inducible expression of *IFNB1* mRNA in pathways mediated by the recognition of dsRNA and other PAMPs.

### 3.6. PCIF1 Deficiency Promoted IFNB1 mRNA Induction at the Transcription Level

As PCIF1 is the only m^6^Am modification enzyme specific to the m^7^G cap, its loss eliminates m^6^Am modification adjacent to the mRNA cap [20,21,22,23]. It has been reported that m^6^Am modification regulates the transcription, stability, and translation of modified mRNA [33,47,48,49,50,51]. Furthermore, we have previously shown that PCIF1 suppresses the expression of reporter genes driven by various transcriptional activation domains [41] and is recruited to a wide range of gene promoters transcribed by RNA polymerase II (Pol II) in a transcription-dependent manner [52]. Therefore, we investigated whether the enhancement in poly(I:C)-induced type I IFN gene mRNA expression caused by PCIF1 deficiency was due to changes in transcription and/or mRNA stability.

**Figure 6:**
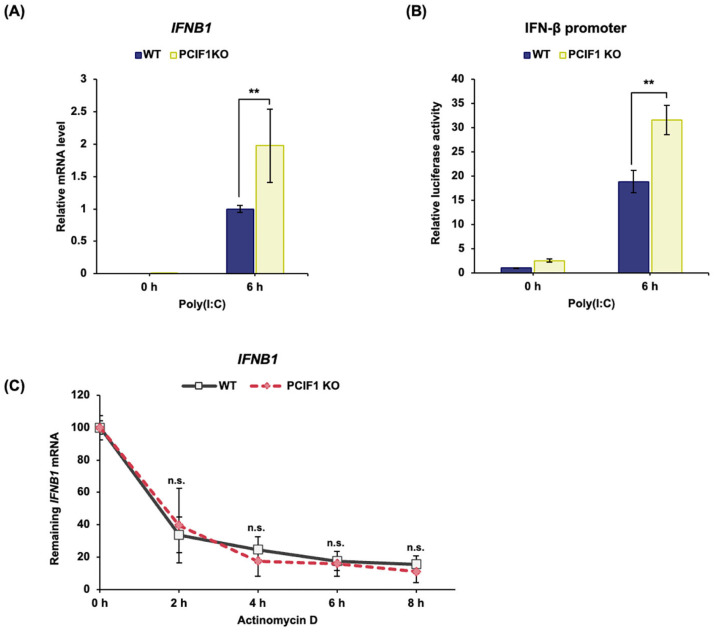
PCIF1 deficiency promoted *IFNB1* mRNA induction at the transcription level. (**A**) WT and PCIF1 KO A549 cells were stimulated with poly(I:C), and nascent RNA was labeled with 5-ethynyl uridine (EU) 6 h after stimulation. Total RNA was isolated from the cells, and labeled RNA was biotinylated using the click reaction. Biotinylated RNA was purified using streptavidin beads. Purified RNA was analyzed by RT-qPCR using a specific primer set for *IFNB1* amplification. (**B**) WT and PCIF1 KO#2 HEK293T cells were co-transfected with the IFN-β-Luc reporter plasmid and the internal control *Renilla* luciferase vector phRG-B for 24 h, followed by stimulation with poly (I:C) for 6 h. Relative luciferase activities were normalized to that of the WT unstimulated control (0 h). (**C**) WT and PCIF1 KO A549 cells were treated with actinomycin D to inhibit transcription. The cells were harvested at 0, 2, 4, 6, and 8 h after treatment, and total RNA was isolated. The amount of residual *IFNB1* mRNA was analyzed by RT-qPCR at each time point using a specific primer set for *IFNB1* mRNA. Relative values were calculated using the expression level of β-actin mRNA (*ACTB*) as the normalization factor. The relative values at each time point were calculated with respect to the time of zero. Data are presented as mean ± SD from three independent experiments. Statistical significance was determined using a two-tailed *t*-test (n.s., *p* > 0.05; ** *p* < 0.01).

To investigate whether PCIF1 regulates *IFNB1* transcription, newly synthesized RNA was pulse-labeled with 5-EU and purified after poly(I:C) stimulation, and nascent *IFNB1* RNA levels were examined using RT-qPCR. The result showed that a marked increase in newly synthesized *IFNB1* mRNA levels was observed in poly (I:C)-stimulated PCIF1 KO A549 cells compared to WT cells (Figure 6A). These findings suggest that the effect of PCIF1 deficiency on *IFNB1* expression may target the transcriptional process of *IFNB1* expression. To further examine this, a dual-luciferase assay was performed to evaluate the impact of PCIF1 deficiency on the expression of reporter genes driven by the IFN-β promoter. As a result, a significant enhancement in IFN-β promoter activity induced by poly(I:C) stimulation was observed in PCIF1 KO A549 cells (Figure 6B). We also investigated whether PCIF1 deficiency affects IFN-β mRNA stability. WT or PCIF1 KO A549 cells were treated with actinomycin D to inhibit transcription after poly(I:C) stimulation, and RNA was collected at various time points. The remaining mRNA levels were analyzed by RT-qPCR. The results showed that the decay rate of *IFNB1* mRNA after transcriptional inhibition was almost the same in PCIF1 KO and WT A549 cells (Figure 6C). Taken together, these results suggest that PCIF1 suppresses *IFNB1* expression at the transcriptional level rather than through mRNA stability regulation.

### 3.7. PCIF1 Deficiency Promotes Phosphorylation and Nuclear Import of IRF3

**Figure 7:**
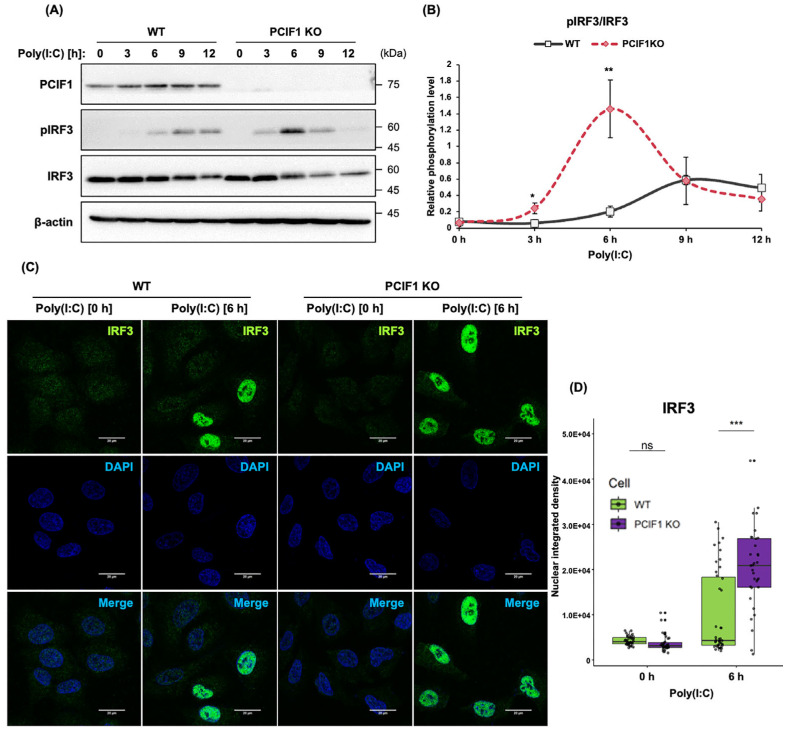
PCIF1 deficiency promotes phosphorylation and nuclear import of IRF3. (**A**) A549 cells were stimulated with poly(I:C), and the cells were harvested at different time points after stimulation. Immunoblot analysis of total protein extracts from WT and PCIF1 KO A549 cells using the indicated antibodies. (**B**) Signal intensities obtained from the immunoblots were quantified using Fiji software version 1.54p. The intensities of pIRF3 were normalized to that of total IRF3. Data are presented as mean ± SD from three independent experiments. Statistical significance was determined using a two-tailed *t*-test (* *p* < 0.05; ** *p* < 0.01). (**C**) WT and PCIF1 KO A549 cells were fixed and immunostained with anti-IRF3 mAb (green), followed by stimulation with poly (I:C) for 6 h. Nuclei were stained with DAPI (blue). Images were acquired using a confocal fluorescence microscope. Scale bars, 20 μm. (**D**) The fluorescence intensity of IRF3 in the nucleus was quantified using Fiji software version 1.54p. Data are presented as mean ± SD (WT at 0 h, *n* = 40; WT at 6 h, *n* = 43; PCIF1 KO at 0 h, *n* = 32; PCIF1 KO at 6 h, *n* = 33). Statistical significance was determined using a two-way ANOVA with Tukey’s test (ns, *p* > 0.05; *** *p* < 0.001).

In type I IFN response, the transcriptional induction of *IFNB1* and *IFNA1* is mainly driven by the activation of the transcription factor IRF3 [6,53,54,55]. Signal transduction initiated by the recognition of PAMPs by cellular sensors leads to the phosphorylation of IRF3 via the cytoplasmic kinase TBK1 [53,54,55]. After forming a homodimer, IRF3 translocates to the nucleus and activates the transcription of the target genes. Therefore, we investigated whether the enhancement in type I IFN gene induction resulting from PCIF1 deficiency was due to its effect on IRF3 activation. As the phosphorylation of Ser386 in IRF3 is critical for the transcriptional activation of target genes [56], we used the level of this phosphorylation as a marker for IRF3 activation. In WT A549 cells, the phosphorylation of IRF3 increased 6 h after poly(I:C) stimulation and peaked at 9 h after stimulation. In contrast, in PCIF1 KO A549 cells, this phosphorylation was observed as early as 3 h after stimulation, peaking at 6 h. Furthermore, compared to WT, PCIF1 deficiency also led to enhanced IRF3 phosphorylation (Figure 7A,B). We further evaluated the effect of PCIF1 on the nuclear translocation of IRF3 by immunostaining. Upon poly(I:C) stimulation, nuclear translocation of IRF3 was observed in both WT and PCIF1 KO A549 cells (Figure 7C). Notably, the amount of IRF3 localized to the nucleus in PCIF1 KO A549 cells was significantly higher than that in WT cells, indicating that nuclear translocation of IRF3 was promoted in PCIF1-deficient cells (Figure 7C,D). Collectively, these results suggest that PCIF1 negatively regulates the transcription of type I IFN genes at least in part by suppressing IRF3 phosphorylation and its nuclear translocation. Alternatively, PCIF1 is a protein localized within the cell nucleus [52] and exhibits transcriptional repression activity [41]. Therefore, it cannot be excluded that PCIF1 may play a direct role in the transcription of *IFNB1*. This will be discussed further in the Section 4.

### 3.8. PCIF1 Regulates Type I IFN Responses in an RNA Methylation Activity-Independent Manner

**Figure 8:**
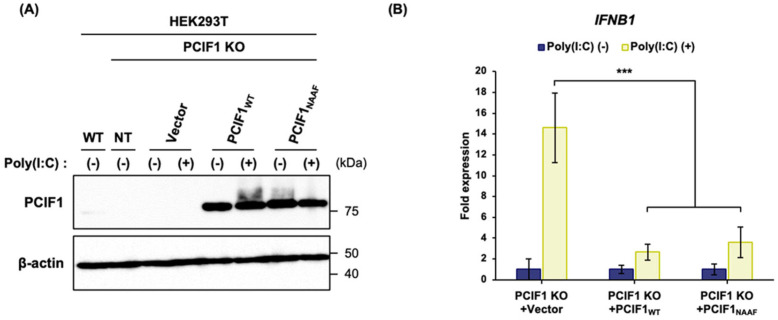
PCIF1 regulates type I IFN responses in an RNA methylation activity-independent manner. (**A**,**B**) PCIF1 KO HEK293T cells were transfected with a control empty vector (Vector), a vector expressing wild-type PCIF1 (PCIF1_WT_), or a methyltransferase-deficient mutant PCIF1 (PCIF1_NAAF_) for 24 h, followed by poly(I:C) stimulation for 6 h. (**A**) Immunoblot analysis of total protein extracts from WT and PCIF1 KO HEK293T cells overexpressing Vector, PCIF1_WT_, or PCIF1_NAAF_ using anti-PCIF1 and anti-β-actin antibodies. (**B**) RT-qPCR analysis of total RNA isolated from PCIF1 KO HEK293T cells overexpressing Vector, PCIF1_WT_, or PCIF1_NAAF_ using a specific primer set for *IFNB1* mRNA. Data are presented as mean ± SD from three independent experiments. Statistical significance was determined using a two-tailed *t*-test (*** *p* < 0.001).

Next, we investigated whether the enhanced poly(I:C)-induced *IFNB1* expression caused by PCIF1 deficiency could be rescued by introducing exogenous PCIF1 into the cells. Furthermore, we used a methyltransferase-inactive mutant to examine whether PCIF1-mediated suppression of *IFNB1* induction depended on RNA methyltransferase activity. Specifically, we employed a methyltransferase-inactive mutant in which the conserved NPPF motif, critical for methyl group transfer within the PCIF1 catalytic domain, was replaced with NAAF [42]. First, we overexpressed either wild-type PCIF1 or a methyltransferase-inactive mutant in PCIF1 KO 293T cells via transfection (Figure 8A). We then analyzed poly(I:C)-induced *IFNB1* expression using RT-qPCR. As expected, overexpression of the wild-type PCIF1 significantly suppressed *IFNB1* expression (PCIF1 KO + PCIF1_WT_; Figure 8B). Interestingly, the expression of the methyltransferase-inactive mutant also suppressed *IFNB1* induction to a similar extent as the wild-type (PCIF1 KO + PCIF1_NAAF_; Figure 8B). These results suggest that PCIF1 negatively regulates type I IFN induction, independent of RNA methyltransferase activity.

## 4. Discussion

This study reveals a novel role for PCIF1 as a negative regulator of type I IFN induction, operating through a mechanism independent of its canonical RNA methyltransferase activity. Our findings demonstrate that PCIF1 deficiency significantly enhances poly(I:C)-induced type I IFN and ISG expression, which is associated with enhanced STAT1 activation and accelerated IRF3 phosphorylation and nuclear translocation. Crucially, we identified this suppressive function at the transcriptional level, offering a critical new perspective on PCIF1’s multifaceted cellular roles beyond epitranscriptomics, particularly in innate immunity.

In this study, we demonstrated that PCIF1 negatively regulates the transcription of type I IFN genes, at least by inhibiting the phosphorylation and nuclear translocation of IRF3. However, the precise mechanisms by which PCIF1 exerts these inhibitory effects remain to be elucidated. The suppression of IRF3 phosphorylation by PCIF1 may involve the inhibition of phosphorylation by kinases such as TBK1 [57]; the promotion of dephosphorylation by IRF3 phosphatases such as PP2A [58], PP1 [59], and MPK5 [60]; or influence on upstream pathways. Furthermore, PCIF1 may affect the stability of IRF3, its nuclear localization, or its interaction with transcriptional coactivators [61]. Consequently, future investigations are warranted to determine whether PCIF1 is involved in regulating these functions.

Regarding the molecular mechanism of transcriptional repression of type I IFN genes by PCIF1, it is possible that not only the inhibition of IRF3 activation is involved but also that PCIF1’s ability to bind to phosphorylated Pol II CTD via its WW domain contributes to its repressive function [20,40]. The CTD consists of tandem repeats of a heptad sequence (YSPTSPS), and the fifth serine residue of the heptad is phosphorylated (pSer5) by TFIIH at the onset of transcription initiation, offering pSer5 as a general feature of transcription initiation [62]. Importantly, our previous work has shown that PCIF1 can specifically bind to pSer5-CTD via its WW domain [20,40], dynamically locate to gene promoters in a transcription-dependent manner [52], and suppress transcriptional activation of reporter genes through this interaction [41,63]. Intriguingly, studies on the fly ortholog of mammalian PCIF1 have shown that it loses RNA methyltransferase activity but retains the ability to specifically bind pSer5-CTD via its WW domain [25,38]. Moreover, Franco et al. showed that fly Pcif1 deficiency led to increased expression of genes involved in mitochondrial ATP synthesis, suggesting that fly Pcif1 may also function as a transcriptional suppressor [38]. These findings suggest that PCIF1 may attenuate excessive transcriptional activation by binding to the Pol II-CTD through its WW domain at gene promoters, thereby affecting transcription initiation, pause release, or elongation independent of its RNA methyltransferase activity. This attenuation could occur through several mechanisms. For instance, PCIF1 binding might sterically hinder key transcription factors or co-activators from interacting with Pol II, or it could promote the recruitment of transcriptional co-repressors or chromatin modifiers, thereby altering local chromatin accessibility or the efficiency of Pol II recruitment. Alternatively, its interaction with pSer5-CTD might stabilize a paused Pol II state or interfere with the transition from the paused state to productive elongation.

While our models speculate that PCIF1 may function as a transcriptional repressor by a general mechanism, an important question on the mechanism by which PCIF1 specifically suppresses a subset of genes involved in type I IFN induction remains unresolved. This specificity could potentially arise from unique promoter architectural features shared among IFN-related genes or from the presence of specific cofactors that bridge PCIF1 to these promoters. For example, PCIF1 might interact with uncharacterized transcription factors or chromatin remodelers that are selectively recruited to IFN gene regulatory elements, thereby targeting its repressive activity. Future studies should aim to identify the direct molecular targets or binding partners of PCIF1 in the context of type I IFN induction, perhaps through proteomic screens or targeted biochemical assays. In addition, beyond the WW domain, detailed mutagenesis studies could systematically identify other domains within PCIF1 (e.g., potential interaction domains for co-factors or chromatin modifiers) that contribute to its transcriptional-repressive function. Rescue assays with these mutants would then pinpoint the critical regions for its non-catalytic role in innate immunity.

Although Tartell et al. demonstrated that PCIF1 could attenuate IFN-β-mediated antiviral effects through RNA methyltransferase activity [34], our findings revealed a distinct methyltransferase-independent mechanism by which PCIF1 broadly suppresses type I IFN induction by directly regulating *IFNB1* transcription and IRF3 activation. This suggests a more fundamental role for PCIF1 as an innate immune checkpoint, potentially acting upstream of specific m^6^Am-mediated viral or host mRNA modifications. This observed dual functionality of PCIF1, a broad methyltransferase-independent transcriptional repression of *IFNB1* and its reported m^6^Am-dependent fine-tuning of specific viral or host transcripts [34,35,36], suggests sophisticated, multi-layered regulation of innate immunity. Non-catalytic transcriptional repression could generally attenuate excessive IFN production, thereby preventing immunopathology. In contrast, its catalytic activity might offer a more precise, context-dependent modulation of specific transcripts, potentially adapting the host response to particular viral threats or cellular states. This raises intriguing questions about how these two distinct functions are coordinated and prioritized during an antiviral response, as well as whether they are independently regulated or converge on common pathways.

## 5. Conclusions

In conclusion, this study fundamentally redefines PCIF1’s role in innate immunity by establishing it as a novel, methyltransferase-independent suppressor of type I IFN induction. Our findings demonstrate that PCIF1 attenuates IRF3 phosphorylation and nuclear translocation, leading to the transcriptional repression of *IFNB1* expression, thereby critically modulating the antiviral response. Given PCIF1’s role as a negative regulator of the type I IFN pathway, its modulation presents an exciting therapeutic avenue. For instance, inhibiting the repressive function of PCIF1 might enhance antiviral immunity in chronic viral infections where IFN responses are dampened. Conversely, activating or mimicking PCIF1’s suppressive role could be beneficial in autoimmune diseases. Future drug discovery efforts could focus on identifying small molecules that modulate PCIF1’s protein–protein interactions, particularly those involving its WW domain interaction with Pol II CTD, rather than its enzymatic activity.

## 6. Limitations

While our study robustly demonstrated a methyltransferase-independent role for PCIF1 in modulating type I IFN responses, it had some limitations. Our investigations were primarily conducted in immortalized cell lines (293T and A549), which may not fully recapitulate the complexity of immune responses in primary cells or under physiologically relevant conditions. Future studies using primary immune cells or animal models of viral infection are crucial to validate these findings and assess the therapeutic potential of targeting PCIF1 in various viral infection and autoimmune disease models.

## Figures and Tables

**Table 1:** Pathway analysis of the upregulated genes from DEGs.

Pathway Name	*p*-Value	Genes
Interferon alpha/beta signaling	3.77 × 10^−4^	*EGR1*; *IFI6*; *OASL*; *IFIT2*
Regulation of cortical dendrite branching	6.68 × 10^−4^	*ROBO1*
MECP2 regulates transcription factors	4.03 × 10^−3^	*MEF2C*
Heme signaling	4.31 × 10^−3^	*MED1*; *MEF2C*; *HMOX1*
Regulation of commissural axon pathfinding by SLIT and ROBO	5.73 × 10^−3^	*ROBO1*
RND3 GTPase cycle	7.76 × 10^−3^	*DLG5*; *ARHGAP5*; *RND3*
Interferon signaling	1.16 × 10^−2^	*EGR1*; *IFI6*; *UBE2L6*; *OASL*; *IFIT2*
Chondroitin sulfate biosynthesis	2.30 × 10^−2^	*CSGALNACT2*; *CSPG4*
Assembly of active LPL and LIPC lipase complexes	3.21 × 10^−2^	*FGF21*
Glutamate neurotransmitter release cycle	3.61 × 10^−2^	*SLC1A1*; *SLC1A3*

## Data Availability

The data presented in this study are openly available in the Gene Expression Omnibus at https://www.ncbi.nlm.nih.gov/geo/query/acc.cgi?acc=GSM4743694 (accessed on 30 September 2023) [GSE156768].

## Supplementary Materials

The following supporting information can be downloaded at https://www.mdpi.com/article/10.3390/cells15030303/s1. Figure S1: Characterization of poly(I:C) stimulation in 293T cells; Figure S2: Characterization of poly(I:C) stimulation in A549 cells, Figure S3: PCIF1 suppresses *IFNB1* induction upon poly(I:C) stimulation; Figure S4: PCIF1 suppresses *IFNB1* induction upon LPS and R848 stimulation; Supplemental Document S1: Supplemental figure legends, Supplemental experimental procedures including primer sequences and antibodies. Table S1: A list of differentially expressed genes upon PCIF1 suppression.
