# Supplementary material for: PCIF1 Attenuates Type I Interferon Induction by Inhibiting IRF3 Activation in a Methyltransferase-Independent Manner
Source: Cells. 2026 Feb 5;15(3):303. doi: 10.3390/cells15030303 (PMC12896973; doi:10.3390/cells15030303)
Supplement: Supplementary file 1 [file cells-15-00303-s001.zip › Supplemental document S1.pdf]

Supplemental Figure legends

Figure S1. Characterization of poly(I:C) stimulation in HEK293T cells.

(A) 293T cells were stimulated with poly(I:C), and the cells were harvested at indicated the time points. Immunoblot analysis of total protein extracts from 293T cells was performed using the indicated antibodies. (B) Signal intensities obtained from the immunoblots were quantified using the FIJI software. The intensities of PCIF1 were normalized to that of β-actin. Data are presented as means±SD from three independent experiments. Statistical significance was determined using a two-tailed t-test (n.s., $P > 0.05$).

Figure S2. Characterization of poly(I:C) stimulation in A549 cells.

(A) Growth curves of WT and PCIF1 KO A549 cells cultured under normal conditions. (B) A549 cells were stimulated with poly(I:C) and harvested at the indicated time points. Immunoblot analysis of total protein extracts from A549 cells using the indicated antibodies. (C) Signal intensities obtained from the immunoblots were quantified using the FIJI software. The intensities of PCIF1 were normalized to β-actin. Data are presented as means±SD from three independent experiments. Statistical significance was determined using a two-tailed t-test (n.s., $P > 0.05$).

Figure S3: PCIF1 suppresses *IFNB1* induction upon poly(I:C) stimulation.

WT and PCIF1 KO (#1 and #2) 293T cells were stimulated with poly(I:C) for twenty-four hours. RT-qPCR analysis of total RNA isolated from the cells using a specific primer set for *IFNB1* mRNA. Data are presented as means±SD from three independent experiments. Statistical significance was determined using a two-tailed *t*-test (** $P < 0.01$).

Figure S4: PCIF1 suppresses *IFNB1* induction upon LPS and R848 stimulation.

WT and PCIF1 KO A549 cells were stimulated with LPS (A) or R848 (B) and harvested at the indicated time points. RT-qPCR analysis of total RNA isolated from the cells using the specific primer set for *IFNB1* mRNA. Data are presented as means±SD from three independent experiments. Statistical significance was determined using a two-tailed *t*-test (** $P < 0.01$).

Supplemental Experimental Procedures

siRNAs

The siRNAs used in this study were as follows:

siPCIF1#1 sense strand: 5’-AAGUAGUAGGGACGAUUCUCCCUCC-3’

antisense strand: 5'-GGAGGGAGAAUCGUCCCUACUACUU-3'

siPCIF1#2 sense strand: 5'-AAACAUGGAAGGUGACACGACUGGU-3'

antisense strand: 5'-ACCAGUCGUGUCACCUUCCAUGUUU-3'

siNC sense strand: 5'-AUUCUAUCACUGCGUGACUU-3'

antisense strand: 5'-GUCACGCUAGUGAUAGAAUUU-3'

Primers

The RT-PCR primers for mRNAs used in this study were as follows:

PCIF1_F: 5'-AGCTGGCTTCGGAAGGACCAC-3'

PCIF1_R: 5'-GCCACACTGCCTCCGCAGAT-3'

IFI6_F: 5'-TGCGGCAGAAGGCGGTATCG-3'

IFI6_R: 5'-CCGCTGTCCGAGCTCTCCGA-3'

IFI27_F: 5'-GCTACTCTGCAGTCACTGGG-3'

IFI27_R: 5'-GCCCAGGATGAACTTGGTCA-3'

IFIT1_F: 5'-GAGGAGCCTGGCTAAGCAAA-3'

IFIT1_R : 5'-GCTCCAGACTATCCTTGACCTG-3'

IFIT2_F : 5'-GCCGAACAGCTGAGAATTGC-3'

IFIT2_R : 5'-CCGTAGGCTGCTCTCCAAGG-3'

OASL_F : 5'-CATCACGGTCACCATTGTGC-3'

OASL_R: 5'-AAATTTCCAGGACCACCGCA-3'

GAPDH_F: 5'-GCTCTCTGCTCCTCCTGTTC-3'

GAPDH_R: 5'-ACGACCAAATCCGTTGACTC-3'

IFNA1_F: 5'-CTGGGAGGTTGTCAGAGCAG-3'

IFNA1_R: 5'-ATGAAAGCGTGACCTGGTGT-3'

IFNB1_F: 5'-AGCACTGGCTGGAATGAGAC-3'

IFNB1_R: 5'-TCCTTGGCCTTCAGGTAATG-3'

ACTB_F: 5'-AGAAATCTGGCACCACACC-3'

ACTB_R: 5'-TAGCACAGCCTGGATAGCAA-3'

r18S_F: 5'-GTAACCCGTTGAACCCATT-3'

r18S_R: 5'-CCATCCAATCGGTAGTAGCG-3'

Antibodies

The antibodies used in this study were as follows: anti-PCIF1 (developed in our laboratory [33]); anti-β-actin (Sigma-Aldrich, Inc., St. Louis, MO, USA, A5441); anti-pSTAT1 (pY701.4A) (Santa Cruz Biotechnology, Dallas, TX, USA, sc-136229); anti-STAT1 (Cell Signaling Technology, Inc., Danvers, MA, USA, #9172S); anti-IFIT2 (F-12) (Santa Cruz Biotechnology, Dallas, TX, USA, sc-390724); anti-DDDDK-tag (Medical & Biological Laboratories Co., Tokyo, Japan, PM020); anti-pIRF3 (Ser386) (E7JBC) (Cell Signaling Technology, Inc., Danvers, MA, USA, 37829S); anti-IRF3 (Cell Signaling Technology, Inc., Danvers, MA, USA, 11904T); and anti-IRF3 (SL-12) (Santa Cruz Biotechnology, Dallas, TX, USA, sc-136470).
