# Supplementary material for: PCIF1 Attenuates Type I Interferon Induction by Inhibiting IRF3 Activation in a Methyltransferase-Independent Manner
Source: Cells. 2026 Feb 5;15(3):303. doi: 10.3390/cells15030303 (PMC12896973; doi:10.3390/cells15030303)
Supplement: Supplementary file 1 [file cells-15-00303-s001.zip › Supplemental Table S1.pdf]

upregulated genes

Gene Symbol	Gene Title	p-value
EXOC5	exocyst complex component 5	0.008
MAP3K12	mitogen-activated protein kinase kinase kinase 12	0.018
PLEK2	pleckstrin 2	0.018
DLG5	discs, large homolog 5 (Drosophila)	0.018
CSPG4	chondroitin sulfate proteoglycan 4	0.018
EFNB2	ephrin-B2	0.018
FGF21	fibroblast growth factor 21	0.018
NAAA	N-acylethanolamine acid amidase	0.018
ARHGAP23	Rho GTPase activating protein 23	0.018
CSGALNACT2	chondroitin sulfate N-acetylgalactosaminyltransferase 2	0.021
NLRP1	NLR family, pyrin domain containing 1	0.021
C15orf27	chromosome 15 open reading frame 27	0.021
HEATR1	HEAT repeat containing 1	0.022
C19orf12	chromosome 19 open reading frame 12	0.022
C5orf41	chromosome 5 open reading frame 41	0.022
THUMPD1	THUMP domain containing 1	0.023
HMOX1	heme oxygenase (decycling) 1	0.025
C1S	complement component 1, s subcomponent	0.025
TPD52L1	tumor protein D52-like 1	0.028
PTGER4	prostaglandin E receptor 4 (subtype EP4)	0.028
CAMSAP1L1	calmodulin regulated spectrin-associated protein 1-like 1	0.028
PROS1	protein S (alpha)	0.028
ERC1	ELKS/RAB6-interacting/CAST family member 1	0.028
PPPDE1	PPPDE peptidase domain containing 1	0.028
IRF2BP2	interferon regulatory factor 2 binding protein 2	0.028
RND3	Rho family GTPase 3	0.028
FSD1	fibronectin type III and SPRY domain containing 1	0.028
ZNF469	zinc finger protein 469	0.028
MED1	mediator complex subunit 1	0.028
IFI6	interferon, alpha-inducible protein 6	0.028
INHBE	inhibin, beta E	0.028
CSGALNACT2	chondroitin sulfate N-acetylgalactosaminyltransferase 2	0.028
VDR	vitamin D (1,25- dihydroxyvitamin D3) receptor	0.028
CAMSAP1L1	calmodulin regulated spectrin-associated protein 1-like 1	0.028
HIST1H2BD	histone cluster 1, H2bd	0.028
UBR1	ubiquitin protein ligase E3 component n-recognin 1	0.028
C5orf43	chromosome 5 open reading frame 43	0.028
TECPR2	tectonin beta-propeller repeat containing 2	0.028
TRAPPC6B	trafficking protein particle complex 6B	0.028
TIMP2	TIMP metallopeptidase inhibitor 2	0.028
RBM24	RNA binding motif protein 24	0.028
SYNPO2	synaptopodin 2	0.028
CDADC1	cytidine and dCMP deaminase domain containing 1	0.028

CA12	carbonic anhydrase XII	0.029
TMTC1	transmembrane and tetratricopeptide repeat containing 1	0.029
PLAGL2	pleiomorphic adenoma gene-like 2	0.030
HIST1H2BD	histone cluster 1, H2bd	0.030
TBPL1	TBP-like 1	0.030
HOXA5	homeobox A5	0.030
STRBP	spermatid perinuclear RNA binding protein	0.030
TIMP2	TIMP metallopeptidase inhibitor 2	0.030
NSBP1	nucleosomal binding protein 1	0.030
ATP8B2	ATPase, class I, type 8B, member 2	0.031
BIRC7	baculoviral IAP repeat-containing 7	0.031
HKDC1	hexokinase domain containing 1	0.031
FANCF	Fanconi anemia, complementation group F	0.031
PRPH	peripherin	0.031
EGR1	early growth response 1	0.031
ADORA1	adenosine A1 receptor	0.031
PPM1A	protein phosphatase 1A (formerly 2C), magnesium-dependent, alpha isoform	0.031
GNPNAT1	glucosamine-phosphate N-acetyltransferase 1	0.032
REV3L	REV3-like, catalytic subunit of DNA polymerase zeta	0.032
MICALL1	MICAL-like 1	0.033
LMOD3	leiomodin 3 (fetal)	0.033
C16orf81	chromosome 16 open reading frame 81	0.033
HEATR1	HEAT repeat containing 1	0.033
NAT13	N-acetyltransferase 13 (GCN5-related)	0.033
NLRP1	NLR family, pyrin domain containing 1	0.033
RAB23	RAB23, member RAS oncogene family	0.034
KCNH5	potassium voltage-gated channel, subfamily H (eag-related), member 5	0.034
PCCA	propionyl Coenzyme A carboxylase, alpha polypeptide	0.034
ACAP2	ArfGAP with coiled-coil, ankyrin repeat and PH domains 2	0.034
ZNF468	zinc finger protein 468	0.034
HEXIM1	hexamethylene bis-acetamide inducible 1	0.035
EPS15	epidermal growth factor receptor pathway substrate 15	0.035
KCNN4	potassium intermediate/small conductance calcium-activated channel, subfamily N, member 4	0.035
TMTC1	transmembrane and tetratricopeptide repeat containing 1	0.036
HTRA3	HtrA serine peptidase 3	0.037
DRAM	damage-regulated autophagy modulator	0.037
ARHGAP28	Rho GTPase activating protein 28	0.037
CCL5	chemokine (C-C motif) ligand 5	0.037
RNF24	ring finger protein 24	0.037
NT5E	5'-nucleotidase, ecto (CD73)	0.037
CROT	carnitine O-octanoyltransferase	0.037
UBE2L6	ubiquitin-conjugating enzyme E2L 6	0.037
SLC1A1	solute carrier family 1 (neuronal/epithelial high affinity glutamate transporter, system Xag), member 1	0.038
MTFMT	mitochondrial methionyl-tRNA formyltransferase	0.038

PTGES	prostaglandin E synthase	0.040
ABCC4	ATP-binding cassette, sub-family C (CFTR/MRP), member 4	0.041
MUM1	melanoma associated antigen (mutated) 1	0.042
MARCKS	myristoylated alanine-rich protein kinase C substrate	0.043
RAPGEF3	Rap guanine nucleotide exchange factor (GEF) 3	0.043
ACRC	acidic repeat containing	0.043
HRASLS2	HRAS-like suppressor 2	0.043
COPZ2	coatomer protein complex, subunit zeta 2	0.043
FZD4	frizzled homolog 4 (Drosophila)	0.043
IFIT2	interferon-induced protein with tetratricopeptide repeats 2	0.043
TMEM55A	transmembrane protein 55A	0.045
UBR1	ubiquitin protein ligase E3 component n-recognin 1	0.045
CA12	carbonic anhydrase XII	0.045
LRP12	low density lipoprotein-related protein 12	0.045
NOSTRIN	nitric oxide synthase trafficker	0.045
CDC42EP3	CDC42 effector protein (Rho GTPase binding) 3	0.045
PPPDE1	PPPDE peptidase domain containing 1	0.045
AMMECR1	Alport syndrome, mental retardation, midface hypoplasia and elliptocytosis chromosomal region gene 1	0.045
DEFB1	defensin, beta 1	0.046
CLIP4	CAP-GLY domain containing linker protein family, member 4	0.047
MINPP1	multiple inositol polyphosphate histidine phosphatase, 1	0.047
MYO1G	myosin IG	0.048
CA12	carbonic anhydrase XII	0.048
DTNA	dystrobrevin, alpha	0.048
C8orf73	chromosome 8 open reading frame 73	0.049

downregulated genes		
Gene Symbol	Gene Title	p-value
LCN15	lipocalin 15	0.007
RBM12	RNA binding motif protein 12	0.007
VAMP3	vesicle-associated membrane protein 3 (cellubrevin)	0.007
VAMP3	vesicle-associated membrane protein 3 (cellubrevin)	0.009
CLN6	ceroid-lipofuscinosis, neuronal 6, late infantile, variant	0.009
C1orf133	chromosome 1 open reading frame 133	0.011
DERL3	Der1-like domain family, member 3	0.011
CYB5A	cytochrome b5 type A (microsomal)	0.011
C5orf35	chromosome 5 open reading frame 35	0.011
CFL2	cofilin 2 (muscle)	0.011
GSTZ1	glutathione transferase zeta 1	0.011
CITED2	Cbp/p300-interacting transactivator, with Glu/Asp-rich carboxy-terminal domain, 2	0.011
PIGA	phosphatidylinositol glycan anchor biosynthesis, class A	0.012
LYRM5	LYR motif containing 5	0.012
TCP11L1	t-complex 11 (mouse)-like 1	0.012
AKAP12	A kinase (PRKA) anchor protein 12	0.014
TM4SF1	transmembrane 4 L six family member 1	0.014
CDK6	cyclin-dependent kinase 6	0.014
DMD	dystrophin	0.015
EDNRA	endothelin receptor type A	0.015
MARCKSL1	MARCKS-like 1	0.015
PXMP3	peroxisomal membrane protein 3, 35kDa	0.015
COL18A1	collagen, type XVIII, alpha 1	0.015
PLEKHA3	pleckstrin homology domain containing, family A (phosphoinositide binding specific) member 3	0.015
TMEM47	transmembrane protein 47	0.015
ARL6IP6	ADP-ribosylation-like factor 6 interacting protein 6	0.015
TMEM45A	transmembrane protein 45A	0.015
MAFK	v-maf musculoaponeurotic fibrosarcoma oncogene homolog K (avian)	0.015
CFL2	cofilin 2 (muscle)	0.015
CGA	glycoprotein hormones, alpha polypeptide	0.015
FOXL1	forkhead box L1	0.015
NR2E3	nuclear receptor subfamily 2, group E, member 3	0.016
RP5-1022P6.2	hypothetical protein KIAA1434	0.016
FHL1	four and a half LIM domains 1	0.016
CNOT6	CCR4-NOT transcription complex, subunit 6	0.016
PITPNC1	phosphatidylinositol transfer protein, cytoplasmic 1	0.016
SEC23A	Sec23 homolog A (S. cerevisiae)	0.017
HERPUD2	HERPUD family member 2	0.017
STMN3	stathmin-like 3	0.018
SMAD2	SMAD family member 2	0.018
CD24	CD24 molecule	0.019
DERL3	Der1-like domain family, member 3	0.020
SVOPL	SVOP-like	0.020

CFL2	cofilin 2 (muscle)	0.020
UGCGL2	UDP-glucose ceramide glucosyltransferase-like 2	0.020
PERP	PERP, TP53 apoptosis effector	0.020
C17orf61	chromosome 17 open reading frame 61	0.021
OLFM1	olfactomedin 1	0.021
TPD52	tumor protein D52	0.022
PAQR6	progestin and adipoQ receptor family member VI	0.022
TFRC	transferrin receptor (p90, CD71)	0.022
FAM120C	family with sequence similarity 120C	0.023
CITED2	Cbp/p300-interacting transactivator, with Glu/Asp-rich carboxy-terminal domain, 2	0.023
BNIP3	BCL2/adenovirus E1B 19kDa interacting protein 3	0.023
PCIF1	PDX1 C-terminal inhibiting factor 1	0.024
ARL6IP6	ADP-ribosylation-like factor 6 interacting protein 6	0.024
PTPN2	protein tyrosine phosphatase, non-receptor type 2	0.024
TMEM50B	transmembrane protein 50B	0.026
MBP	myelin basic protein	0.027
MOSC1	MOCO sulphurase C-terminal domain containing 1	0.027
LYPD6	LY6/PLAUR domain containing 6	0.028
GM2A	GM2 ganglioside activator	0.028
DEPDC1B	DEP domain containing 1B	0.029
NSDHL	NAD(P) dependent steroid dehydrogenase-like	0.029
RSBN1	round spermatid basic protein 1	0.033
CXCR4	chemokine (C-X-C motif) receptor 4	0.033
VMA21	VMA21 vacuolar H+-ATPase homolog (S. cerevisiae)	0.033
RASSF2	Ras association (RalGDS/AF-6) domain family member 2	0.033
CYB5A	cytochrome b5 type A (microsomal)	0.034
CYB5A	cytochrome b5 type A (microsomal)	0.034
FHL1	four and a half LIM domains 1	0.034
NEXN	nexilin (F actin binding protein)	0.035
TMEM47	transmembrane protein 47	0.035
KIAA0895	KIAA0895	0.038
ALOX5	arachidonate 5-lipoxygenase	0.040
PDE4D	phosphodiesterase 4D, cAMP-specific (phosphodiesterase E3 dunce homolog, Drosophila)	0.043
PVRL2	poliovirus receptor-related 2 (herpesvirus entry mediator B)	0.043
ZC3H6	zinc finger CCCH-type containing 6	0.048
