# Supplementary figures and images for: PCIF1 Attenuates Type I Interferon Induction by Inhibiting IRF3 Activation in a Methyltransferase-Independent Manner
Source: Cells. 2026 Feb 5;15(3):303. doi: 10.3390/cells15030303 (PMC12896973; doi:10.3390/cells15030303)

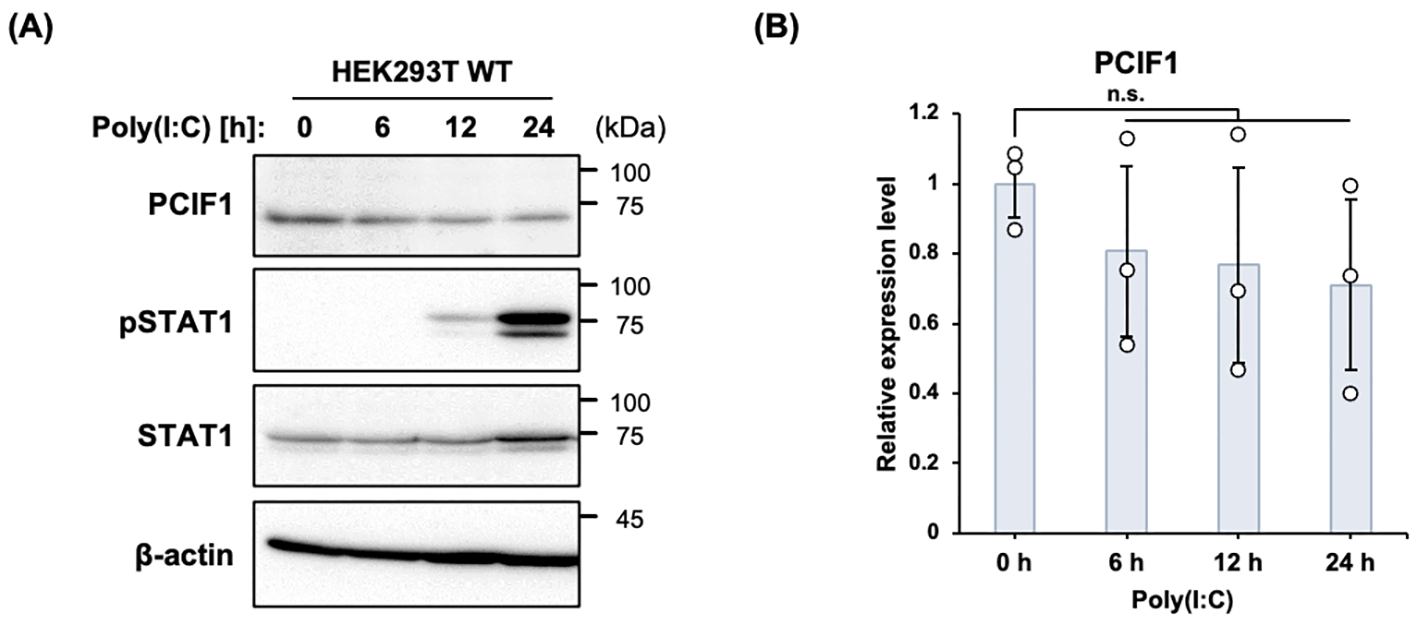

Supplement: Supplementary file 1 [file cells-15-00303-s001.zip › Figure S1.tiff]

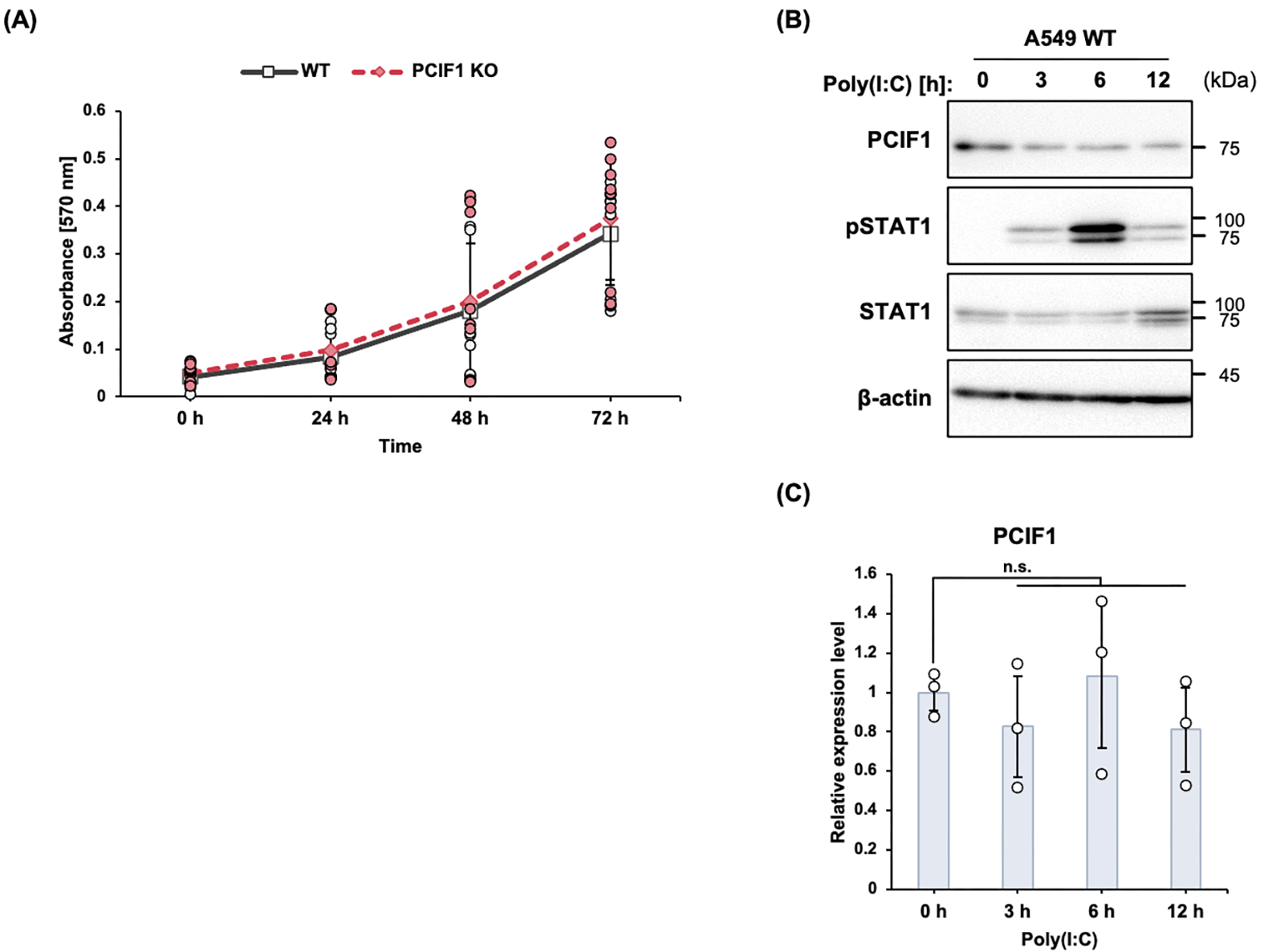

Supplement: Supplementary file 1 [file cells-15-00303-s001.zip › Figure S2.tiff]

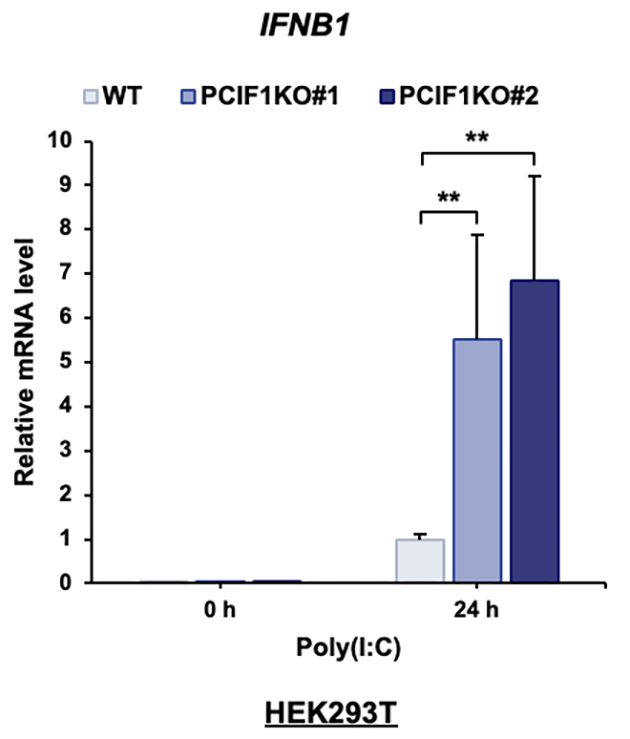

Supplement: Supplementary file 1 [file cells-15-00303-s001.zip › Figure S3.tiff]

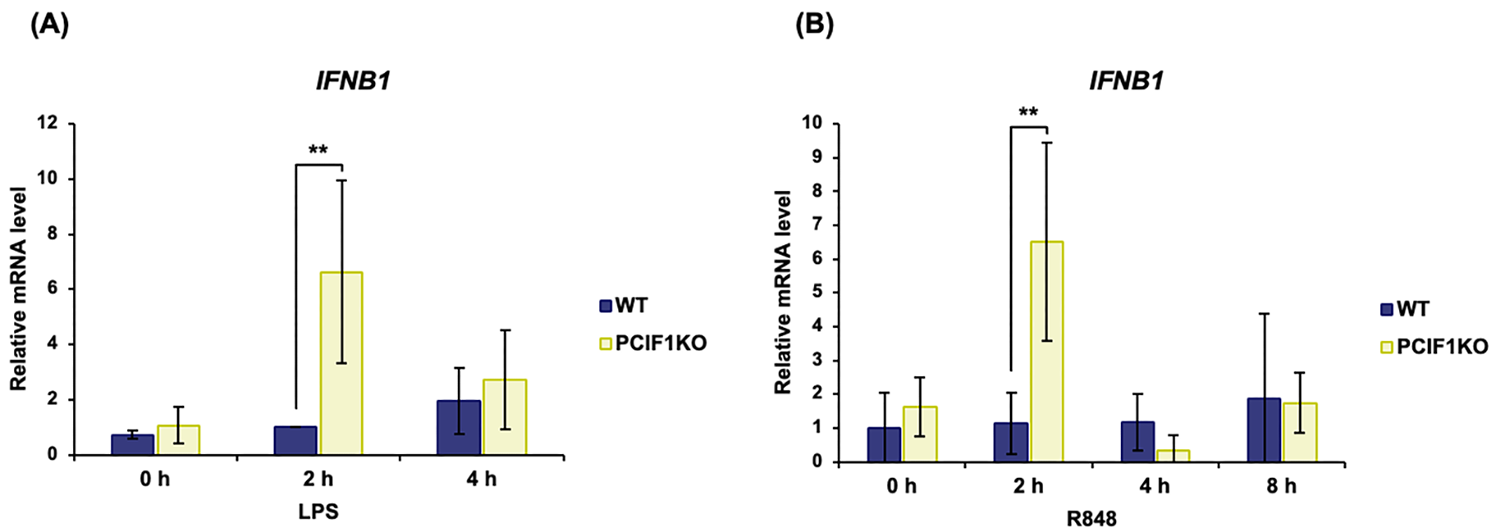

Supplement: Supplementary file 1 [file cells-15-00303-s001.zip › Figure S4.tiff]
